# Uridine Inhibits Hepatocellular Carcinoma Cell Development by Inducing Ferroptosis

**DOI:** 10.3390/jcm12103552

**Published:** 2023-05-18

**Authors:** Liuliu Zi, Wangbin Ma, Lilong Zhang, Boyang Qiao, Zhendong Qiu, Junhui Xu, Jiacheng Zhang, Yahong Ye, Yueyuan Yang, Keshuai Dong, Chen Chen, Weixing Wang, Qingyan Zhao

**Affiliations:** 1 Department of Hepatobiliary and Laparoscopic Surgery, Renmin Hospital of Wuhan University, No. 238, Jiefang Road, Wuchang District, Wuhan 430060, China; 2 Hubei Key Laboratory of Cardiology, Cardiovascular Research Institute of Wuhan University, Renmin Hospital of Wuhan University, Wuhan 430060, China; 3 School of Pharmaceutical Sciences, Wuhan University, Wuhan 430072, China; 4 Department of Endocrinology & Metabolism, Renmin Hospital of Wuhan University, Wuhan 430060, China

**Keywords:** hepatocellular carcinoma, tumor microenvironment, CAD, DHODH, uridine, ferroptosis

## Abstract

Uridine is a key metabolite used as a substrate for the production of DNA, RNA, and glucose, and it is mainly synthesized in the liver. Currently, it is not known whether uridine levels are altered in the tumor microenvironment of patients with hepatocellular carcinoma (HCC) and whether uridine can be a target for tumor therapy. In this study, the detection of genes associated with de novo uridine synthesis, carbamoyl-phosphate synthetase 2, aspartate transcarbamylase, dihydroorotase (CAD) (n = 115), and dihydroorotate dehydrogenase (DHODH) (n = 115) in HCC tissues through tissue microarrays revealed that the expression of CAD and DHODH was higher in tumor compared with paraneoplastic tissues. Next, we collected tumor tissues from surgically resected HCC patients and the corresponding adjacent non-tumor tissues (n = 46) for LC–MS/MS assays. The results showed that the median and interquartile ranges of uridine content in non-tumor and tumor tissues were 640.36 (504.45–807.43) and 484.22 (311.91–626.73) nmol/g, respectively. These results suggest that uridine metabolism is disturbed in HCC patients. To further investigate whether uridine can be used as a tumor-therapeutic target, a series of high concentrations of uridine were incubated with HCC cells in vitro and in vivo. It was observed that uridine dose-dependently inhibited the proliferation, invasion, and migration of HCC cells by activating the ferroptosis pathway. Overall, these results reveal for the first time the range of uridine content in human HCC tissues and suggest that uridine may be a new target for HCC therapy.

## 1. Introduction

Hepatic cancer, as a tumor of high malignancy, currently ranks sixth in incidence and third in lethality among all tumors, and about 90% of cases are those of hepatocellular carcinoma (HCC) [1]. The high energy and nutrient requirements of tumor cells reflect the specificity of the tumor metabolic microenvironment, and tumor metabolic reprogramming can be the driving force of tumors. 

Recent studies have suggested that carcinogenic/antitumor metabolites could activate/inhibit oncogenic signaling and thus promote/mitigate malignant transformation [2]. As a pyrimidine nucleoside, uridine is a key metabolite in a series of essential processes by which the body produces DNA, RNA, proteins, and glucose as an energy reserve [3,4,5]. Studies suggest that uridine is synthesized primarily in the liver, with adipocytes serving as its production reserve [6]. Most cells are incapable of synthesizing uridine themselves. Their basic operations rely on the uridine in the blood released by the liver and adipocytes; meanwhile, uridine is generally maintained within a narrow range in the plasma [7]. The combination of uridine and pyruvate may be used as a potential treatment for disorders of the oxidative phosphorylation system (OXPHOX) [8]. Uridine may alleviate CCl4-induced liver fibrosis [9]. Uridine supplementation may rejuvenate aging stem cells, promoting the regeneration of various tissues in the body [10,11]. The administration of uridine may promote repair after myocardial injury [12]. Uridine is a pyrimidine nucleoside of plant/animal origin and, therefore, can also be taken up and absorbed by the body and accompany the food intake process. Despite its significant physiological role, uridine has received poor attention compared to other nucleosides, such as adenosine. An enhanced understanding of the physiological mechanisms controlling this metabolite has the potential to shed light on several disease states, including cancer. Compared to normal cells, tumor cells require large amounts of DNA and RNA during their continuous division and expansion and, therefore, may have a high demand for uridine. Currently, no studies have focused on the synthesis of uridine in patients with HCC and its effect on HCC cells. Therefore, the study of uridine may be of great importance to our understanding of the mechanism of HCC development.

Uridine synthesis consists of both de novo and salvage synthesis pathways, with quiescent cells relying primarily on the salvage synthesis pathway and proliferating cells (e.g., tumor cells) relying on the de novo synthesis pathway [13]. The rate-limiting enzymes for the de novo synthesis of uridine include dihydroorotate dehydrogenase (DHODH) and carbamoyl-phosphate synthetase 2, aspartate transcarbamylase, dihydroorotase (CAD). CAD is a fusion of three structural domains that encode carbamoyl-phosphate synthetase 2, aspartate transcarbamylase, and dihydroorotase, which are related to de novo uridine synthesis [14]. DHODH is located downstream of CAD and oxidizes dihydroorotate to orotate [15]. In contrast to various other enzymes involved in de novo uridine synthesis, DHODH exists on the external layer of the internal membrane of mitochondria and links de novo uridine synthesis to the mitochondrial electron transport chain [16]. In this study, the correlation between the expression of de novo uridine synthesis genes CAD and DHODH in HCC patients and survival data was investigated for the first time by a clinical histochemical microarray, and the uridine content of tumor tissues of hepatocellular carcinoma patients was examined by mass spectrometry. We also explored the mechanism of action of uridine administration on HCC cells by in vitro administration and the construction of subcutaneous tumors in nude mice.

## 2. Materials and Methods

### 2.1. Patients

This prospective study was conducted on patients recruited from Renmin Hospital of Wuhan University, and all individuals provided informed consent. Tumor tissue and para-carcinoma tissues from 46 patients diagnosed with HCC were collected and then cut into pieces less than one cm^3^, followed by immediate freezing in liquid nitrogen, after which they were stored at −80 °C until LC–MS/MS was conducted. The ethical committee of Renmin Hospital of Wuhan University gave the approval to conduct this study. Human HCC tissues of microarray plates were obtained from 115 patients at Tongji Hospital of Huazhong University of Science and Technology between 2012 and 2016. Data including clinical information and pathological features were collected and were approved by the institutional ethics committee of Huazhong University of Science and Technology. Written informed consent for data analysis was obtained from all patients before operation. To avoid potential heterogeneity, only data on HCC with cirrhosis were included. Participants with other digestive disorders, malignancies, acute or chronic autoimmune diseases, and infectious diseases were excluded. 

### 2.2. LC–MS/MS for Uridine Measurements

The concentration of uridine was detected by LC–MS/MS (AB SCIEX ExionLC and AB SCIEX QTRAP 6500+ mass spectrometer, Applied Biosystems Sciex, Redwood City, CA, USA), and the positive electrospray ionization mode (ESI) was utilized to conduct MS analysis. The data were procured and processed with the help of AB SCIEX Analyst 1.6.3 software (Applied Biosystems Sciex, Redwood City, CA, USA).

### 2.3. Cell Lines and Cell Culture

HEPG2, 97H, and HLF cell lines were obtained from the American Type Culture Collection (ATCC, Manassas, VA, USA). Modified Eagle’s medium comprising penicillin (100 U/mL), streptomycin (100 U/mL), and 10% fetal bovine serum (Gibco, Carlsbad, CA, USA) was utilized for cell culture. Cells used in subsequent experiments were all between the 4th and 8th generations.

### 2.4. Calculation of Cell Viability and Cell Death Percentage

The CCK8 kit (BioSharp, Hefei, China) was utilized for measuring cell activity. The cells were inoculated in 96-well plates at a cell density of 3000 cells/well. Uridine at concentrations of 0, 30, 150, 300, and 600 μM was added, and estimation of OD values was done at 450 nm. The cell death percentage was measured with an Annexin V-FITC apoptosis assay kit, calculated as per the guidelines provided by the manufacturer, and evaluated using a Beckman Coulter CytoFLEX S flow cytometer.

### 2.5. Transwell and Wound Healing

Transwell 1 × 10^5^/mL cells were suspended in 200 uL culture medium without Gibco in the upper chamber of a 24-well plate (pore size 8 μm; Corning), and 500 μL culture medium constituting 10% Gibco was introduced into the lower chamber. The stained cells were counted under a Nikon light microscope (Nikon Corporation, Tokyo, Japan) after being co-cultured for 24 h. Three random unduplicated visual fields were captured for analysis under 200× microscopy. The experiment was performed in triplicate. Wound healing experiments: Sterile pipette tips were used to scratch the monolayer cells, after which PBS was employed to wash the cells thrice, followed by incubation in a serum-free medium for 24 h. Photographs were collected under the Nikon light microscope (Nikon Corporation) at 40× field of view for analysis at 0 and 24 h. The area difference, as well as mobility, were calculated using ImageJ 1.8.0 software.

### 2.6. Measurement of ROS

Intracellular ROS were detected with the peroxide-sensitive fluorescent probe DCFH-DA. Fluorescence intensity was detected by Beckman Coulter (Brea, CA, USA), CytoFLEX S flow cytometry analysis. FlowJo or Graphpad Prism 8.0 software was used to evaluate the data.

### 2.7. Malondialdehyde (MDA) and GSH Assay

MDA, the main indicator of lipid peroxidation, was measured with the kit as per the guidelines provided by the manufacturer and normalized by cell number. The Glutathione Assay Kit (#G2026-1000T) (Servicebio, Wuhan, China) was used to assess GSH concentration, which was normalized by protein concentration as per the guidelines provided by the manufacturer.

### 2.8. Immunohistochemistry

A fluorescent microscope (Olympus BX63, Tokyo, Japan) was used to take immunohistochemistry images. Five images were taken randomly of each section for each immunostaining index, and their mean values were calculated for statistical comparison. Digital image analysis was performed with ImageJ software to quantitatively characterize the integrated optical density (IOD) of immunostained sections. The high or low expression group was defined according to their medians. All data were expressed as mean ± standard deviation. SPSS 21.0 statistical software (SPSS Inc., Chicago, IL, USA) was utilized to conduct statistical analysis of data.

### 2.9. Hematoxylin and Eosin (HE) Staining

Subcutaneous tumors removed from animal models of HCC in different treatment groups were fixed in a 4% paraformaldehyde solution, followed by dehydration using alcohol and decolorization using xylene detergent. The transparent tissues were embedded in wax, cut using a microtome, and affixed to carrier glasses. This was followed by dewaxing and staining of the cells with hematoxylin (H) dye and eosin € dye (AB245880, Abcam, Cambridge, UK). Once again, the dehydration of cells was done using alcohol, and they were decolorized using xylene detergent. The tissues were sealed and then photographed for observation using the optical microscope.

### 2.10. Quantitative Reverse Transcription PCR (qRT-PCR)

Calculation of the relative gene expression levels was done with the help of the 2-ΔΔCT method. GAPDH was used as a normalized internal reference. The primer sequences are mentioned below: GAPDH-F:TCGGAGTCAACGGATTTGGT;GAPDH-R:TTCCCGTTCTCAGCCTTGAC, DHODH-F:CCACGGGAGATGAGCGTTTC, DHODH-R:CAGGGAGGTGAAGCGAACA, CAD-F:GCCAGCCCAGACAAATTACCTATACC, CAD-R:AGACGCCAGAGCCAAGGACTAG.

### 2.11. In Vivo Tumor Model

The animal studies were approved by the Ethics Committee of the Wuhan University People’s Hospital and conformed to the Guidelines for the Care and Use of Laboratory Animals of the Ministry of Science and Technology of the People’s Republic of China. The digested 97 h cells were washed 3 times with cold PBS to achieve a final concentration of 2.5 × 106/mL. One hundred μL of cell suspension was administered via a subcutaneous route into the right dorsal side of BALB/c male nude mice. Random allotment of the mice into 3 groups was performed: control group, uridine 100 mg/kg group, and uridine 300 mg/kg group. A total of six mice were allotted to each group, and then we marked the sites of mice that inoculated cells. One hundred µL of PBS was subcutaneously injected into the control group at the same location daily. Uridine was injected locally starting from the second day of cell suspension injection. The samples were collected, and mice were executed after 16 consecutive days of injection.

### 2.12. Statistical Analysis

Data are shown in the forms of median ^(2^5th^–7^5th percentiles) or mean ± SD. A two-sample independent *t*-test was used for comparison between two groups, and ANOVA tests were used between multiple groups. A paired t-test was used to analyze the uridine content and gene expression in tumor tissues and the paired adjacent tissues. * *p* < 0.05, ** *p* < 0.01, and *** *p* < 0.001 were considered statistically significant. 

## 3. Results

### 3.1. Upregulation of the De Novo Uridine Synthesis Pathway in Patients with HCC

**Figure 1:**
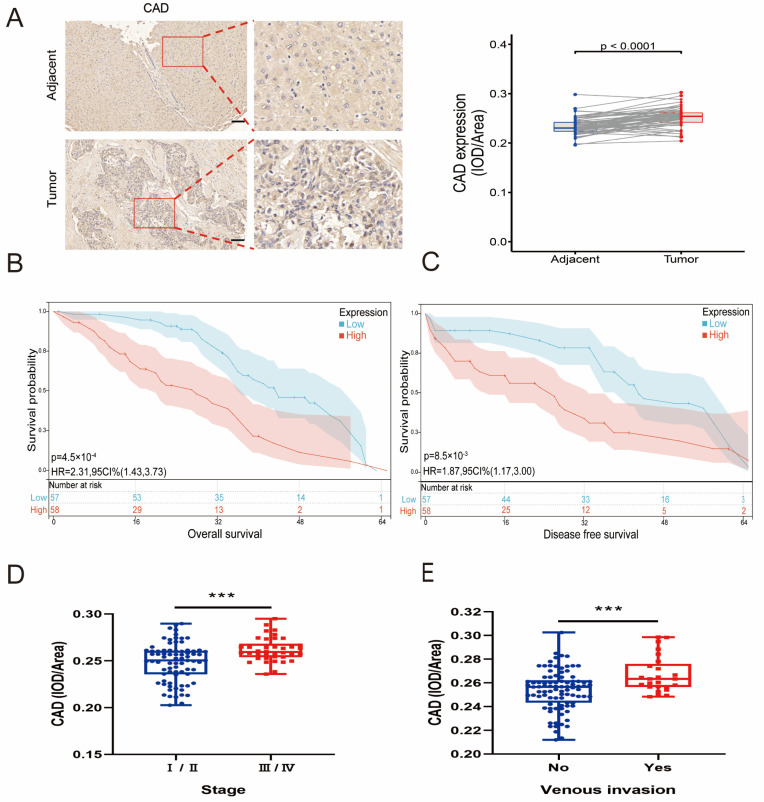
Preliminary verification of CAD signature in HCC. (**A**) Typical images of CAD immunohistochemistry in HCC and quantitative analysis of CAD expression. The images show a 16× magnification. Scale bar = 50 μm. (**B**) Relationship between CAD expression and OS. (**C**) Relationship between CAD expression and DFS. (**D**) Relationship between CAD expression in HCC histochemical microarray and pathological stage. (**E**) Relationship between CAD expression in HCC histochemical microarray and vascular invasion (*** *p* < 0.001). Abbreviations: Adjacent = adjacent noncancerous tissues; IOD = integrated optical density; HR = hazard ratio; OS = overall survival; DFS = disease free survival.

**Figure 2:**
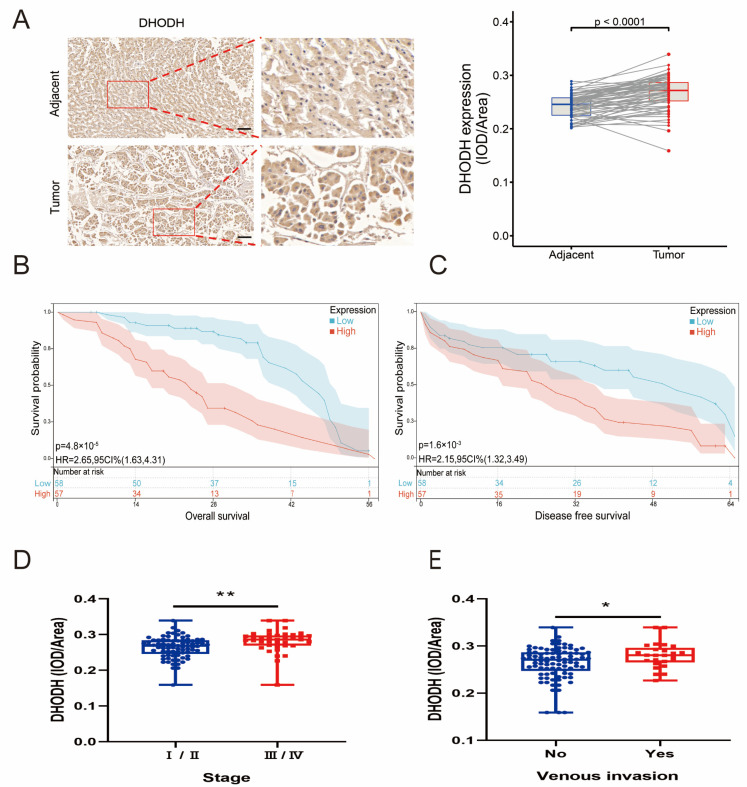
Preliminary verification of DHODH signature in HCC. (**A**) Typical images of DHODH immunohistochemistry in HCC patient tissueand quantitative analysis of DHODH expression. The images show a 16× magnification. Scale bar = 50 μm. (**B**) Relationship between DHODH expression and OS. (**C**) Relationship between DHODH expression and DFS. (**D**) Relationship between DHODH expression in HCC histochemical microarray and pathological stage. (**E**) The relationship between DHODH expression in HCC histochemical microarray and vascular invasion (* *p* < 0.05; ** *p* < 0.01). Abbreviations: Adjacent = adjacent noncancerous tissues; IOD = integrated optical density; HR = hazard ratio; OS = overall survival; DFS = disease free survival.

To investigate the de novo uridine synthesis pathway, CAD expression in serial tissue microarray (TMA) sections containing HCC tissue (n = 115) was examined and matched with adjacent liver tissue (n = 115) using immunohistochemical (IHC) staining. Specifically, most HCC samples showed strong CAD coloring (Figure 1A). Patients with higher CAD expression levels had a shorter overall survival (OS) rate than patients with lower CAD expression (Figure 1B). Patients with higher CAD expression levels had a shorter DFS rate than patients with lower CAD levels (Figure 1C). Patients with high CAD expression usually had higher clinical-stage tumors, indicating a positive correlation of CAD expression levels with the clinical stage of HCC patients (Figure 1D). Patients with higher CAD expression appeared more vulnerable to vascular invasion (Figure 1E). The clinical data from 115 HCC patients revealed a correlation between CAD expression levels and tumor size, Child–Pugh Class, and tumor differentiation (Table S1). DHODH expression, clinical stage, vascular invasion, OS, and DFS in HCC tissues (n = 115) and their corresponding adjacent liver tissues (n = 115) were detected by TMA. It was found that, as with CAD, DHODH expression appeared significantly higher in HCC tissues compared to paraneoplastic tissues, and it showed a positive correlation with clinical stage, vascular invasion, OS, and DFS of HCC (Figure 2A–E). The clinical data from 115 HCC patients revealed the correlation between DHODH expression levels and Child–Pugh Class (Table S2). Therefore, it was indicated that the de novo uridine synthesis pathway is upregulated in patients with HCC.

### 3.2. Disturbance of Uridine Metabolism in the Tumor Microenvironment of HCC

**Figure 3:**
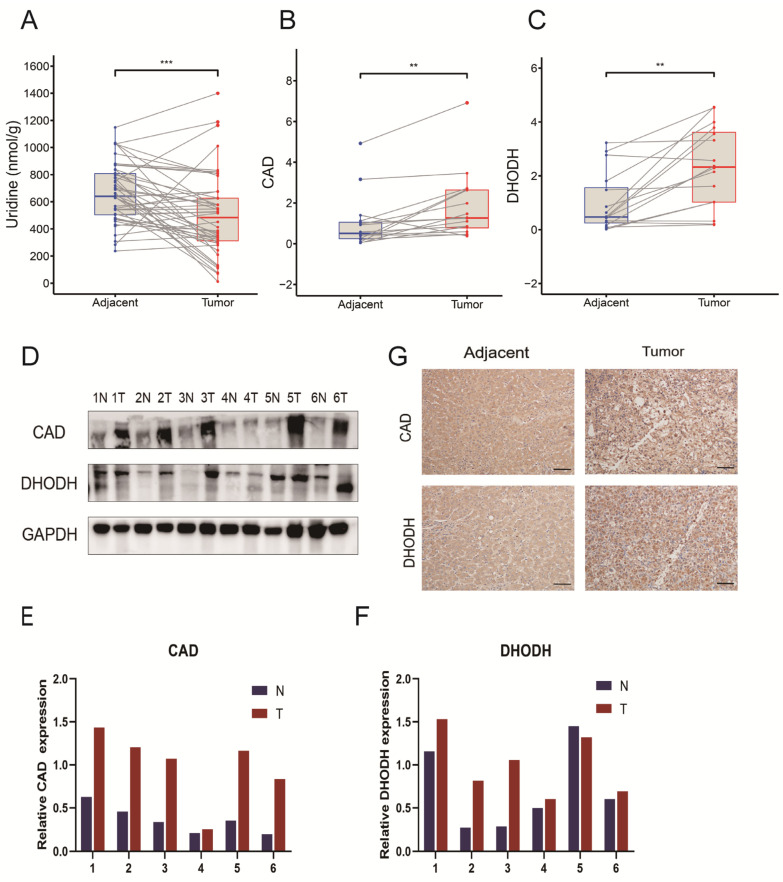
Disturbance in uridine metabolism in the tumor microenvironment of HCC patients. (**A**) Uridine levels in tumor and adjacent noncancerous tissues of HCC patients (n = 46). (**B**,**C**) CAD and DHODH expression in mRNA of tumor and adjacent noncancerous tissues in 16 randomly selected pairs of liver cancer patients. (**D**) The expression of protein CAD and DHODH in tumor and adjacent noncancerous tissues of 6 pairs of liver cancer patients were randomly extracted. (**E**,**F**) The protein quantification of CAD and DHODH in tumor and adjacent noncancerous tissues. (**G**) Typical images of DHODH and CAD immunohistochemistry in patients with HCC. Scale bar = 50 μM. (** *p* < 0.01; *** *p* < 0.001). Abbreviations: Adjacent = adjacent noncancerous tissues.

Uridine is a downstream metabolite of CAD and DHODH. To detect the downstream metabolite uridine, uridine levels in tumor tissues from patients with confirmed HCC (n = 46) and their matched adjacent liver tissues (n = 46) were also analyzed. The results showed that the median and interquartile ranges of uridine content in non−tumor and tumor tissues were 640.36 (504.45–807.43) and 484.22 (311.91–626.73) nmol/g, respectively. Notably, the uridine content in liver cancer tissues was significantly lower than that in non-tumor tissues. (Figure 3A). The clinical data from 46 HCC patients revealed a correlation between uridine levels and Child–Pugh Class, vascular invasion, and TNM stage (Table S3). Next, mRNA levels were examined in the tumors and their adjacent liver tissues of 16 randomly selected pairs of HCC patients. DHODH and CAD expressions appeared greater in tumor tissues compared to adjacent tissues of HCC patients and were statistically significant (Figure 3B,C). Similarly, a total of six pairs of HCC patients whose tumors and adjacent liver tissues were tested for protein levels, as well as representative images of histochemistry for DHODH and CAD, were selected, and elevated expressions of DHODH and CAD were observed (Figure 3D–G). These findings indicated a disturbance in uridine metabolism in HCC patients. 

### 3.3. Inhibition of HCC Development by Uridine In Vivo and In Vitro

**Figure 4:**
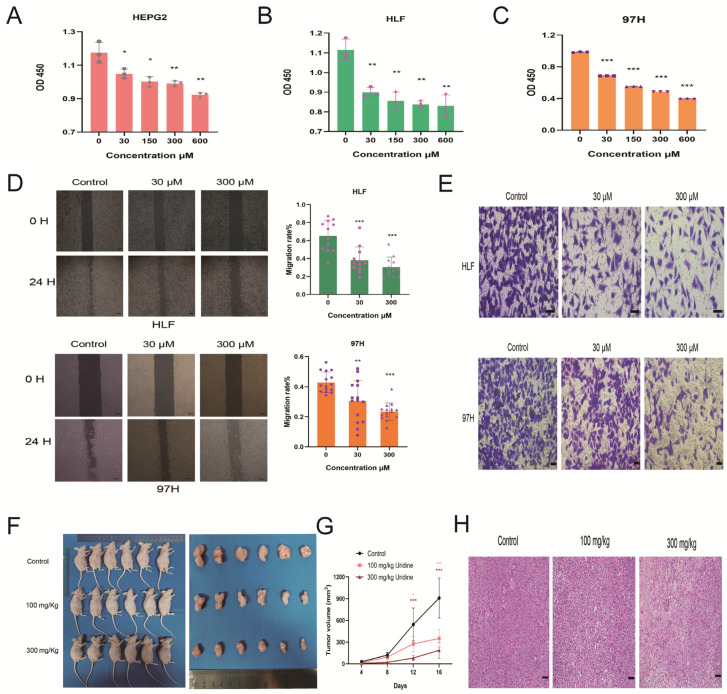
Inhibition of HCC cells’ development by uridine in vitro and in vivo. (**A**–**C**) Different uridine concentrations inhibited the proliferation ability of HEPG2, HLF, and 97H, as determined by CCK8 assay. (**D**) Inhibition of cell migration ability by uridine at 30 and 300 μm concentrations. Magnification, ×40. Scale bar: 200 μm. (**E**) Reduction of invasive ability of HCC cells by 30 and 300 μM concentrations of uridine. Magnification, ×200. Scale bar: 50 μm. (**F**) Images of nude mice bodies and tumor masses at day 16 after transplantation of 97H cells. (**G**) Tumor volume. (**H**) Representative HE images of subcutaneous tumors. Magnification, ×200. Scale bar: 50 μm (* *p* < 0.05; ** *p* < 0.01; *** *p* < 0.001).

For the purpose of investigating uridine’s impact on HCC cells, three HCC cell lines (HEPG2, HLF, and 97H) were used for analysis. Because uridine in the physiological state generally remains within a narrow range (6–8 μM) in plasma [7], we chose a gradient concentration maximum of 100 times higher than the physiological state uridine concentration (0–600 μM). After treatment with uridine at different gradient concentrations for 48 h, the three cell lines were assayed for cell proliferation by the CCK8 assay, and the findings indicated a notable dose-dependent inhibition of the proliferating capability of the three HCC cells (Figure 4A–C). On the basis of these findings, HLF and 97H were selected, which performed with superior effectiveness, for the follow-up experiments. Uridine’s impact on the HLF and 97H HCC cells’ ability to migrate was examined by wound healing assay, and the results showed that uridine caused inhibition of HCC cells’ ability to migrate in a dose-dependent way (Figure 4D). Uridine’s impact on the invasion ability of HLF and 97H HCC cells was examined using Transwell assay, and it was found that uridine caused a dose-dependent inhibition of the invasion ability of HCC cells (Figure 4E). For the purpose of in vivo investigation of uridine’s antitumor effect, 97H cells of nude mice were inoculated by a subcutaneous route for the development of a subcutaneous transplantation tumor model. Both doses of uridine significantly reduced the tumor size of subcutaneous tumors in HCC nude mice dose-dependently in comparison to the control group (Figure 4F–H). To conclude, uridine resulted in a dose-dependent inhibition of HCC growth in both in vivo and in vitro environments.

### 3.4. Inhibition of In Vivo and In Vitro HCC Development by Uridine via Activation of Ferroptosis Pathway

**Figure 5:**
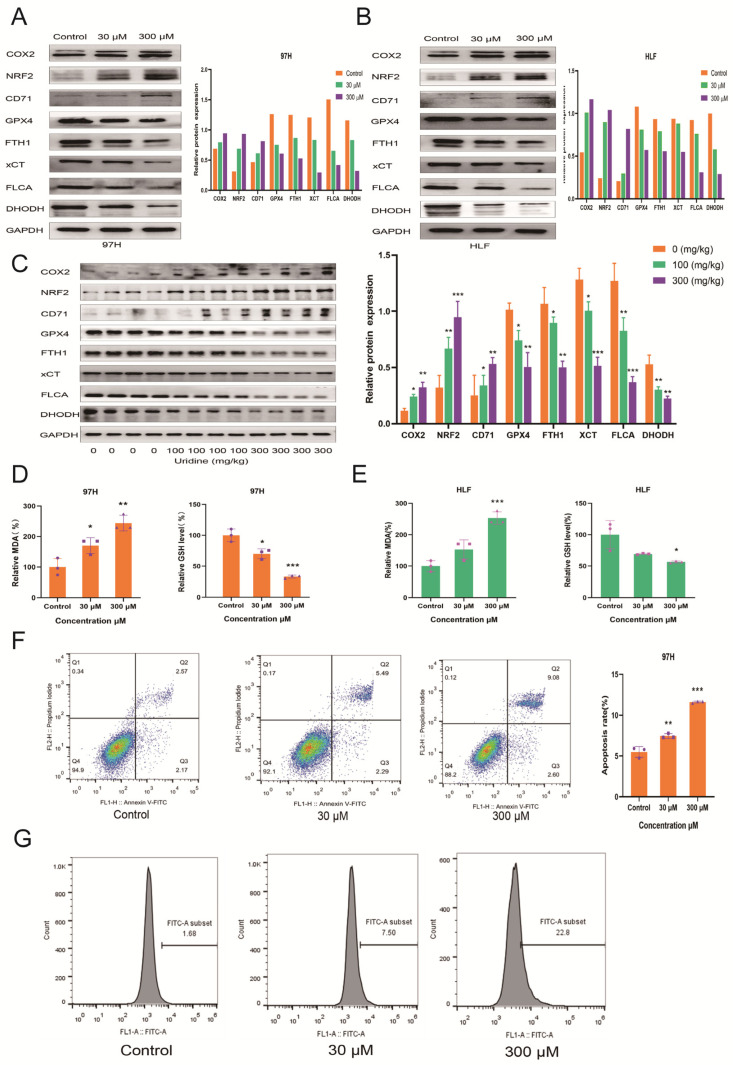
Inhibition of HCC cell development by in vitro uridine treatment via activation of the ferroptosis pathway. (**A**) Immunoblots of COX2, NRF2, CD71, GPX4, FTH1, xCT, FLCA, DHODH with control, 30, and 300 μM uridine concentrations in 97H cells. (**B**) Immunoblots of COX2, NRF2, CD71, GPX4, FTH1, xCT, FLCA, DHODH in HLF cells with control, 30, and 300 μM concentrations of uridine groups. (**C**) Immunoblots of COX2, NRF2, CD71, GPX4, FTH1, xCT, FLCA, DHODH in the subcutaneous tumor with control, 100 mg/kg, and 300 mg/kg concentrations of uridine groups after sampling in nude mice. (**D**) MDA and GSH contents with control, 30, and 300 μM uridine concentrations in 97H cells. (**E**) MDA and GSH contents in HLF cells with control, 30, and 300 μM uridine concentrations. (**F**) Apoptosis rate of 97H cells with control, 30, and 300 μM uridine concentrations. (**G**) ROS production with control, 30, and 300 μM uridine concentrations in 97H cells. (* *p* < 0.05; ** *p* < 0.01; *** *p* < 0.001).

Because DHODH expression was reduced after in vitro and in vivo treatment of HCC cells with uridine, it has been reported as a new mechanism of ferroptosis [17]. To check whether uridine exerts antitumor activity through ferroptosis, the changes in ferroptosis indices after the treatment of HCC cells were examined with two different concentrations of uridine in HLF and 97H cells. After 48 h of uridine treatment, the ferroptosis markers glutathione peroxidase 4 (GPX4), cystine/glutamate reverse transporter protein (xCT), ferritin heavy chain (FTH1), and ferritin light chain (FLCA) at the protein level were downregulated dose-dependently, and then COX2, nuclear factor E2-related factor 2 (NRF2), and CD71 were increased dose-dependently (Figure 5A,B). Furthermore, the association of tumor weight reduction with ferroptosis was also investigated. In comparison to the control group, the ferroptosis-related proteins COX2, NRF2, and CD71 were upregulated, and GPX4, xCT, FTH1, and FLCA were downregulated dose-dependently in the uridine-treated group (Figure 5C), indicating that uridine could inhibit HCC tumor growth in vivo via the induction of ferroptosis. In addition, the lipid peroxidation product MDA and the endogenous antioxidant glutathione GSH were also investigated in both 97H and HLF cell lines, and it was observed that MDA levels were upregulated and GSH levels were depleted dose-dependently (Figure 5D,E). Annexin V-FITC apoptosis assay revealed a dose-dependent increase in apoptosis after the uridine treatment of 97H cells for 48 h (Figure 5F), and the results were statistically significant. In addition, changes in intracellular ROS levels in 97H cells after 48 h of uridine treatment were detected with an ROS fluorescent probe, and flow cytometry results showed that intracellular ROS levels increased with an increase in uridine concentration, indicating that uridine can induce the accumulation of intracellular reactive oxygen species (Figure 5G). Overall, the data show that uridine can cause in vivo and in vitro induction of ferroptosis in HCC cells.

### 3.5. Inhibition of the In Vitro Effect of Uridine on HCC Cells by Ferrostatin-1

**Figure 6:**
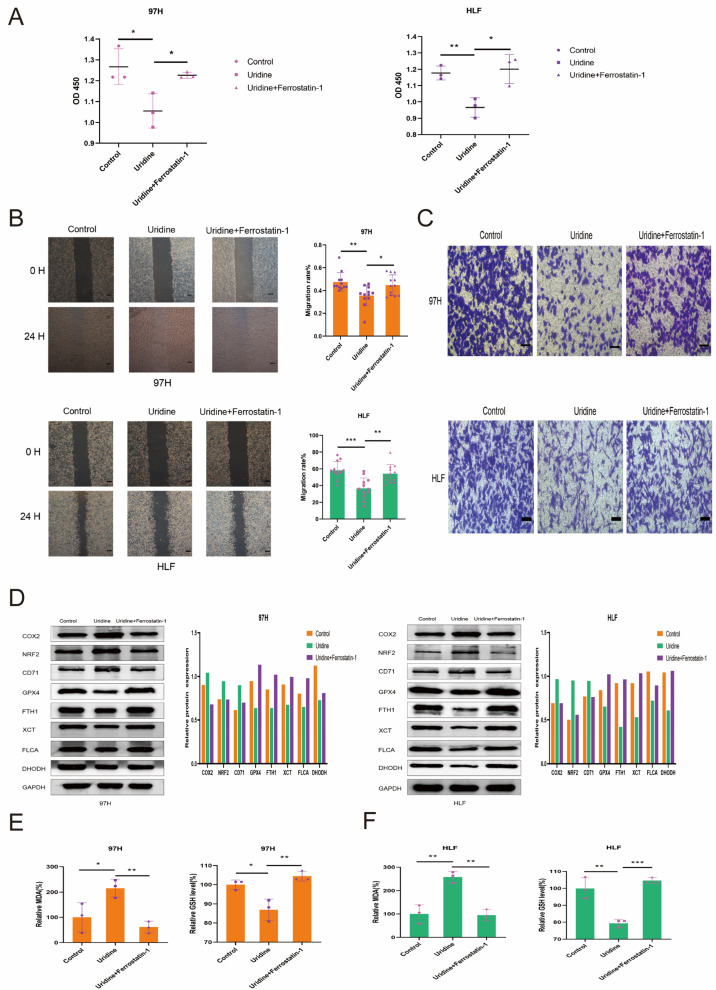
In vitro inhibition of uridine’s impact on HCC cells by Ferrostatin-1, a ferroptosis inhibitor. (**A**) Proliferation ability of control, uridine (30 μM), and uridine (30 μM) + Ferrostatin-1 (1 μM) groups in HLF and 97H cells. (**B**) Control, uridine (30 μM), and uridine (30 μM) + Ferrostatin-1 (1 μM) groups in HLF and 97H cells were examined for cell migration ability. Magnification, ×40. Scale bar: 200 μm. (**C**) Invasive ability of HCC cells in control, Uridine (30 μM), and uridine (30 μM) + Ferrostatin-1 (1 μM) groups in HLF and 97H cells. Magnification, ×200. Scale bar: 50 μm. (**D**) Immunoblotting of COX2, NRF2, CD71, GPX4, FTH1, xCT, FLCA, DHODH in control, uridine (30 μM), and uridine (30 μM) + Ferrostatin-1 (1 μM) groups of HLF and 97H cells. (**E**) MDA and GSH contents in control, uridine (30 μM), and uridine (30 μM) + Ferrostatin-1 (1 μM) groups of 97H cells. (**F**) MDA and GSH contents in HLF cells in control, uridine (30 μM), and uridine (30 μM) + Ferrostatin-1 (1 μM) groups. (* *p* < 0.05; ** *p* < 0.01; *** *p* < 0.001).

It was observed that the ferroptosis inhibitor Ferrostatin-1 blocked the effect of uridine in vitro. A total of three groups based on 97H and HLF cells were set up, which included the control group (DMSO only), uridine group (30 μM), and uridine with Ferrostatin-1 group (30 μM uridine, 1 μM Ferrostatin−1), respectively. The findings showed that the Ferrostatin-1 addition prominently reduced the uridine’s inhibitory action on the proliferation, migration, and invasion of HCC cells (Figure 6A–C). Ferroptosis-related proteins COX2, NRF2, and CD71 were downregulated, and GPX4, xCT, FTH1, and FLCA were upregulated in the uridine and Ferrostatin-1 groups compared to the uridine group and were not statistically different from the control group (Figure 6D). The changes in lipid peroxidation products MDA and endogenous antioxidant glutathione GSH were examined in both 97H and HLF cell lines after treatment with Ferrostatin-1, a ferroptosis inhibitor, and it was observed that MDA levels were downregulated and GSH levels were increased compared with the uridine group treatment (Figure 6E,F). The above results suggest that the ferroptosis inhibitor can alleviate the inhibitory effect of uridine on HCC cells in vitro.

## 4. Discussion

Oral uridine is used to alleviate the dose-limiting leukopenia of 5-fluorouracil (5-FU) in oncology treatment [18], but the effect of uridine itself on tumor cells has not been studied. This research made the unprecedented observation that the presence of uridine disorders in the tumor microenvironment and high local concentrations of uridine inhibit tumor cell development in patients with HCC. The expression of the de novo uridine synthesis genes CAD and DHODH was significantly increased and positively correlated with OS, DFS, clinical stage, and vascular invasion in HCC patients. However, local tumor area uridine levels were reduced. Because of the reduced uridine content in the tumor microenvironment, our experiments have verified that high uridine concentrations can cause inhibition of the division, infiltration, and migration of HCC cells by activating the ferroptosis pathway in vitro and in vivo.

Pyrimidine synthesis has recently been identified as a new therapeutic target in KRAS/LKB1 mutant non-small cell lung cancer and PTEN mutant cancers [19,20]. Growing evidence has shown that targeted nucleotide metabolism, such as pyrimidine synthesis, could promote an antitumor response to immunotherapy. Hence, further investigation of this metabolic reprogramming of pyrimidine anabolic upregulation in patients with HCC could provide therapeutic information and improve outcomes in the development of HCC. The major enzymes involved in pyrimidine synthesis are CAD and DHODH. CAD is a fusion of three structural domains that encode carbamoyl-phosphate synthetase 2, aspartate transcarbamylase, and dihydroorotase, which are related to pyrimidine biosynthesis [14]. Previous studies have found that targeting the AKT2-CAD-mediated pyrimidine synthesis pathway provides a new target for the treatment of HCC [21]. DHODH is located downstream of CAD and oxidizes dihydroorotate to orotate [15]. In contrast to various other enzymes in ab initio pyrimidine synthesis, DHODH exists on the external layer of the internal membrane of mitochondria and links nucleotide synthesis to the mitochondrial electron transport chain [16]. Substances that inhibit DHODH, such as the metabolite teriflunomide, are utilized in the form of immunomodulatory drugs in autoimmune disorders, such as rheumatoid/psoriatic arthritis and multiple sclerosis [22]. The combination of DHODH inhibitors with other ferroptosis-inducing therapeutic agents, such as radiotherapy and immunotherapy, has direct translational implications for cancer treatment [23,24,25,26]. Previous studies have found that either inhibiting CAD [21,27] or DHODH [17], or direct targeting of pyrimidine metabolism [28,29], could inhibit tumor progression. Further, a positive correlation with stage, vascular invasion, DFS, and OS in patients with HCC was observed in combination with clinical information in our histochemical microarray. Thus, it has been demonstrated that CAD and DHODH are closely related to hepatocarcinogenesis and development.

Tumors contain various metabolite drivers or tumor suppressors, and tumor-related metabolite studies are vital for understanding tumorigenesis and progression. The downstream metabolites of CAD and DHODH include uridine, which, as a pyrimidine nucleoside, is a key metabolite in a number of essential processes such as the body’s manufacture of DNA, RNA, and proteins, and the storage of glucose as an energy reserve [3]. Despite its key physiological role, uridine has received limited attention compared to other nucleosides, such as adenosine. The main site of uridine synthesis is the liver, and the effect of uridine on HCC is unknown. Studying uridine may be of great significance for the development of HCC treatments.

Cao et al. verified in UPP1 knockout mice that uridine boosting promotes systemic tumorigenesis and has DNA-damaging effects on healthy cells, both indicating that pharmacological uridine may not be safe [30]. However, Liu et al. subsequently found that long-term oral (7 months) or intraperitoneal (5 months) uridine treatment was non-tumorigenic [10], and these two opposing findings suggest that uridine tumorigenicity needs to be further validated. Neither study further investigated the metabolism of uridine in tumor patients and the effect of uridine on tumor cell development. We have therefore extended their work to investigate the presence of uridine disorders in liver cancer patients themselves and the inhibition of the development of liver cancer cells. It was observed that a high in vitro concentration of uridine could inhibit DHODH expression. Previous studies have shown that DHODH, as a newly discovered third mechanism of ferroptosis, is accompanied by an increase in uridine content after the application of a ferroptosis activator in tumor cells. They also showed that DHODH inhibition induces ferroptosis in GPX4 low cancer cells and sensitizes GPX4 high cancer cells to ferroptosis [17]. Ferroptosis is characterized as a type of regulated cell death induced by excess lipid peroxidation and is a key mechanism of tumor suppression [31,32,33]. GPX4 [34,35], ferroptosis suppressor protein 1 (FSP1) [36,37], and DHODH [17] constitute the major ferroptosis defense system. Therefore, it was speculated and validated that uridine may inhibit HCC cell development by activating the ferroptosis pathway in HCC cells.

Our description of the reduced levels of uridine released into the tumor microenvironment in patients with HCC raises interesting questions for future studies. What are the implications of reduced levels of released uridine in HCC patients for organs that are severely dependent on plasma uridine (e.g., the heart)? Uridine has recently been determined to be a key anti-aging metabolite [10], and it is also worth investigating whether reduced levels of released uridine in patients with HCC have an impact on the degree of aging in other organs throughout the body. To conclude, this study reported that uridine homeostasis is disturbed in patients with HCC and that high uridine concentrations inhibit HCC cell development. HCC reprograms its pyrimidine metabolism to promote self-renewal and tumorigenesis.

## 5. Conclusions

Uridine disorders exist within the tumor microenvironment of HCC patients, and high concentrations of uridine inhibit HCC cell development in vitro and in vivo through the ferroptosis pathway.

## 6. Patents

Our study was approved by the Clinical Research Ethics Committee of Wuhan University People’s Hospital (The number of ethical approval: WDRY2021-KS029).

## Data Availability

The data that support the findings of this study are available from the corresponding author upon reasonable request.

## Supplementary Materials

The following supporting information can be downloaded at: https://www.mdpi.com/article/10.3390/jcm12103552/s1. Table S1: The correlation between CAD expression and clincopathological features in HCC; Table S2: The correlation between DHODH expression and clincopathological features in HCC; Table S3: The correlation between Uridine level and clincopathological features in HCC.

## References

[1] Sung H., Ferlay J., Siegel R.L., Laversanne M., Soerjomataram I., Jemal A., Bray F. (2021). Global Cancer Statistics 2020: GLOBOCAN Estimates of Incidence and Mortality Worldwide for 36 Cancers in 185 Countries. CA Cancer J. Clin..

[2] Ward P.S., Thompson C.B. (2012). Metabolic Reprogramming: A Cancer Hallmark Even Warburg Did Not Anticipate. Cancer Cell.

[3] Yamamoto T., Koyama H., Kurajoh M., Shoji T., Tsutsumi Z., Moriwaki Y. (2011). Biochemistry of uridine in plasma. Clin. Chim. Acta.

[4] Le T.T., Urasaki Y., Pizzorno G. (2014). Uridine prevents tamoxifen-induced liver lipid droplet accumulation. BMC Pharmacol. Toxicol..

[5] Le T.T., Ziemba A., Urasaki Y., Hayes E., Brotman S., Pizzorno G. (2013). Disruption of uridine homeostasis links liver pyrimidine metabolism to lipid accumulation. J. Lipid Res..

[6] Deng Y., Wang Z.V., Gordillo R., An Y., Zhang C., Liang Q., Yoshino J., Cautivo K.M., De Brabander J., Elmquist J.K. (2017). An adipo-biliary-uridine axis that regulates energy homeostasis. Science.

[7] Connolly G.P., Simmonds H., Dulay J.A. (1996). Pyrimidines and CNS regulation: Changes in the levels of pyrimidines may lead to abnormal neurological activity. Trends Pharmacol. Sci..

[8] Adant I., Bird M., Decru B., Windmolders P., Wallays M., de Witte P., Rymen D., Witters P., Vermeersch P., Cassiman D. (2022). Pyruvate and uridine rescue the metabolic profile of OXPHOS dysfunction. Mol. Metab..

[9] Zheng W.V., Li Y., Cheng X., Xu Y., Zhou T., Li D., Xiong Y., Wang S., Chen Z. (2021). Uridine alleviates carbon tetrachloride-induced liver fibrosis by regulating the activity of liver-related cells. J. Cell. Mol. Med..

[10] Liu Z., Li W., Geng L., Sun L., Wang Q., Yu Y., Yan P., Liang C., Ren J., Song M. (2022). Cross-species metabolomic analysis identifies uridine as a potent regeneration promoting factor. Cell Discov..

[11] Ye J., Jin Z., Chen S., Guo W. (2022). Uridine relieves MSCs and chondrocyte senescence *in vitvo* and exhibits the potential to treat osteoarthritis in vivo. Cell Cycle.

[12] Li S., Yokota T., Wang P., Hoeve J.T., Ma F., Le T.M., Abt E.R., Zhou Y., Wu R., Nanthavongdouangsy M. (2022). Cardiomyocytes disrupt pyrimidine biosynthesis in nonmyocytes to regulate heart repair. J. Clin. Investig..

[13] Villa E., Ali E.S., Sahu U., Ben-Sahra I. (2019). Cancer Cells Tune the Signaling Pathways to Empower de Novo Synthesis of Nucleotides. Cancers.

[14] Lane A.N., Fan T.W.-M. (2015). Regulation of mammalian nucleotide metabolism and biosynthesis. Nucleic Acids Res..

[15] Evans D.R., Guy H.I. (2004). Mammalian Pyrimidine Biosynthesis: Fresh Insights into an Ancient Pathway. J. Biol. Chem..

[16] Jones M.E. (1980). Pyrimidine Nucleotide Biosynthesis in Animals: Genes, Enzymes, and Regulation of UMP Biosynthesis. Annu. Rev. Biochem..

[17] Mao C., Liu X., Zhang Y., Lei G., Yan Y., Lee H., Koppula P., Wu S., Zhuang L., Fang B. (2021). DHODH-mediated ferroptosis defence is a targetable vulnerability in cancer. Nature.

[18] van Groeningen C.J., Peters G.J., Pinedo H.M. (1993). Reversal of 5-fluorouracil-induced toxicity by oral administration of uridine. Ann. Oncol..

[19] Mathur D., Stratikopoulos E., Ozturk S., Steinbach N., Pegno S., Schoenfeld S., Yong R., Murty V.V., Asara J.M., Cantley L.C. (2017). PTEN Regulates Glutamine Flux to Pyrimidine Synthesis and Sensitivity to Dihydroorotate Dehydrogenase Inhibition. Cancer Discov..

[20] Kim J., Hu Z., Cai L., Li K., Choi E., Faubert B., Bezwada D., Rodriguez-Canales J., Villalobos P., Lin Y.-F. (2017). CPS1 maintains pyrimidine pools and DNA synthesis in KRAS/LKB1-mutant lung cancer cells. Nature.

[21] Liu F., Gai X., Wu Y., Zhang B., Wu X., Cheng R., Tang B., Shang K., Zhao N., Deng W. (2022). Oncogenic β-catenin stimulation of AKT2–CAD-mediated pyrimidine synthesis is targetable vulnerability in liver cancer. Proc. Natl. Acad. Sci. USA.

[22] O'Connor P., Wolinsky J.S., Confavreux C., Comi G., Kappos L., Olsson T.P., Benzerdjeb H., Truffinet P., Wang L., Miller A. (2011). Randomized Trial of Oral Teriflunomide for Relapsing Multiple Sclerosis. N. Engl. J. Med..

[23] Ye L.F., Chaudhary K.R., Zandkarimi F., Harken A.D., Kinslow C.J., Upadhyayula P.S., Dovas A., Higgins D.M., Tan H., Zhang Y. (2020). Radiation-Induced Lipid Peroxidation Triggers Ferroptosis and Synergizes with Ferroptosis Inducers. ACS Chem. Biol..

[24] Lang X., Green M.D., Wang W., Yu J., Choi J.E., Jiang L., Liao P., Zhou J., Zhang Q., Dow A. (2019). Radiotherapy and Immunotherapy Promote Tumoral Lipid Oxidation and Ferroptosis via Synergistic Repression of SLC7A11. Cancer Discov..

[25] Wang W., Green M., Choi J.E., Gijón M., Kennedy P.D., Johnson J.K., Liao P., Lang X., Kryczek I., Sell A. (2019). CD8^+^ T cells regulate tumour ferroptosis during cancer immunotherapy. Nature.

[26] Lei G., Zhang Y., Koppula P., Liu X., Zhang J., Lin S.H., Ajani J.A., Xiao Q., Liao Z., Wang H. (2020). The role of ferroptosis in ionizing radiation-induced cell death and tumor suppression. Cell Res..

[27] Tu H.-F., Ko C.-J., Lee C.-T., Lee C.-F., Lan S.-W., Lin H.-H., Ku C.-C., Lee D.-Y., Chen I.-C., Chuang Y.-H. (2021). Afatinib Exerts Immunomodulatory Effects by Targeting the Pyrimidine Biosynthesis Enzyme CAD. Cancer Res.

[28] Wang X., Yang K., Wu Q., Kim L.J.Y., Morton A.R., Gimple R.C., Prager B.C., Shi Y., Zhou W., Bhargava S. (2019). Targeting pyrimidine synthesis accentuates molecular therapy response in glioblastoma stem cells. Sci. Transl. Med..

[29] Wu H.-L., Gong Y., Ji P., Xie Y.-F., Jiang Y.-Z., Liu G.-Y. (2022). Targeting nucleotide metabolism: A promising approach to enhance cancer immunotherapy. J. Hematol. Oncol..

[30] Cao Z., Ma J., Chen X., Zhou B., Cai C., Huang D., Zhang X., Cao D. (2016). Uridine homeostatic disorder leads to DNA damage and tumorigenesis. Cancer Lett..

[31] Zhang Y., Shi J., Liu X., Feng L., Gong Z., Koppula P., Sirohi K., Li X., Wei Y., Lee H. (2018). BAP1 links metabolic regulation of ferroptosis to tumour suppression. Nature.

[32] Stockwell B.R., Angeli J.P.F., Bayir H., Bush A.I., Conrad M., Dixon S.J., Fulda S., Gascón S., Hatzios S.K., Kagan V.E. (2017). Ferroptosis: A Regulated Cell Death Nexus Linking Metabolism, Redox Biology, and Disease. Cell.

[33] Jiang L., Kon N., Li T., Wang S.-J., Su T., Hibshoosh H., Baer R., Gu W. (2015). Ferroptosis as a p53-mediated activity during tumour suppression. Nature.

[34] Yang W.S., SriRamaratnam R., Welsch M.E., Shimada K., Skouta R., Viswanathan V.S., Cheah J.H., Clemons P.A., Shamji A.F., Clish C.B. (2014). Regulation of Ferroptotic Cancer Cell Death by GPX4. Cell.

[35] Friedmann Angeli J.P., Schneider M., Proneth B., Tyurina Y.Y., Tyurin V.A., Hammond V.J., Herbach N., Aichler M., Walch A., Eggenhofer E. (2014). Inactivation of the ferroptosis regulator Gpx4 triggers acute renal failure in mice. Nat. Cell Biol..

[36] Doll S., Freitas F.P., Shah R., Aldrovandi M., da Silva M.C., Ingold I., Grocin A.G., da Silva T.N.X., Panzilius E., Scheel C.H. (2019). FSP1 is a glutathione-independent ferroptosis suppressor. Nature.

[37] Bersuker K., Hendricks J.M., Li Z., Magtanong L., Ford B., Tang P.H., Roberts M.A., Tong B., Maimone T.J., Zoncu R. (2019). The CoQ oxidoreductase FSP1 acts parallel to GPX4 to inhibit ferroptosis. Nature.

