# Supplementary material for: Uridine Inhibits Hepatocellular Carcinoma Cell Development by Inducing Ferroptosis
Source: J Clin Med. 2023 May 18;12(10):3552. doi: 10.3390/jcm12103552 (PMC10219358; doi:10.3390/jcm12103552)
Supplement: Supplementary file 1 [file jcm-12-03552-s001.zip › jcm-2212520-supplementary.pdf]

Journal of
Clinical Medicine

MDPI

Supplementary Materials

Table S1. The correlation between CAD expression and clincopathological features in HCC.

Clinical Variables	No. of Patients	CAD Expression		*p* Value
	n = 115	High (n = 57)	Low (n = 58)	
Gender				0.180
Female	44	18 (15.65%)	26 (22.61%)	
Male	71	39 (33.91%)	32 (27.83%)	
Age(years)				0.058
<60	46	28 (24.35%)	18 (15.65%)	
≥60	69	29 (25.22%)	40 (34.78%)	
AFP(ng/ml)				0.456
>400	62	33 (28.70%)	29 (25.22%)	
≤400	53	24 (20.87%)	29 (25.22%)	
ALT(U/L)				0.058
>40	46	28 (25.22%)	18 (14.78%)	
≤40	69	29 (24.35%)	40 (35.65%)	
AST(U/L)				0.852
>40	63	32 (27.83%)	31(26.96%)	
≤40	52	25 (21.74%)	27 (23.48%)	
Child-Pugh Class				**0.049**
A	77	33 (28.70%)	44 (38.26%)	
B	38	24 (20.87%)	14 (12.17%)	
Microvascular invasion				0.056
No	70	40 (34.78%)	30 (26.09%)	
Yes	45	17 (14.78%)	28 (24.35%)	
Tumor size(cm)				**0.041**
≤5	60	24 (20.87%)	36 (31.30%)	
>5	55	33 (28.69%)	22 (19.13%)	
Tumor number				0.703
Multiple	71	34 (29.57%)	37 (32.17%)	
Single	44	23 (20.00%)	21 (18.26%)	
Tumor encapsulation				0.572
No	68	32 (27.83%)	36 (31.30%)	
Yes	47	25 (21.74%)	22 (19.13%)	
Tumor differentiation				**0.041**
Well	56	22 (19.13%)	34 (29.57%)	
Poor	59	35 (30.43%)	24 (20.87%)	
HBsAg				0.268
Negative	14	9 (7.83%)	5 (4.35%)	
Positive	101	48 (41.74%)	53 (46.09%)	
Edmonson grade				0.147
II	64	30 (26.09%)	34 (29.56%)	
III	27	11 (9.57%)	16 (13.91%)	
IV	24	16 (13.91%)	8 (6.96%)	

Table S2. The correlation between DHODH expression and clincopathological features in HCC.

Clinical Variables	No. of Patients	DHODH Expression		*p* Value
	n = 115	High (n = 58)	Low (n = 57)	
Gender				0.703
Female	44	21 (18.26%)	23 (20.00%)	
Male	71	37 (32.17%)	34 (29.57%)	
Age(years)				0.850
<60	46	24 (20.87%)	22 (19.13%)	
≥60	69	34 (29.57%)	35 (30.43%)	
AFP(ng/ml)				0.062
>400	62	26 (22.61%)	36 (31.30%)	
≤400	53	32 (27.83%)	21 (18.26%)	
ALT(U/L)				0.087
>40	46	28 (24.35%)	18 (15.65%)	
≤40	69	30 (26.09%)	39 (33.91%)	
AST(U/L)				0.456
>40	63	34 (29.57%)	29 (25.22%)	
≤40	52	24 (20.87%)	28 (24.35%)	
Child-Pugh Class				**0.010**
A	77	32 (27.83%)	45 (39.13%)	
B	38	26 (22.61%)	12 (10.43%)	
Microvascular invasion				0.446
No	70	33 (28.70%)	37 (32.17%)	
Yes	45	25 (21.74%)	20 (17.39%)	
Tumor size(cm)				0.193
≤5	60	34 (29.57%)	26 (22.61%)	
>5	55	24 (20.87%)	31 (26.96%)	
Tumor number				0.848
Multiple	71	35 (30.43%)	36 (31.30%)	
Single	44	23 (20.00%)	21 (18.26%)	
Tumor encapsulation				0.450
No	68	32 (27.83%)	36 (31.30%)	
Yes	47	26 (22.61%)	21 (18.26%)	
Tumor differentiation				0.265
Well	56	25 (21.74%)	31 (26.96%)	
Poor	59	33 (28.70%)	26 (22.61%)	
HBsAg				0.152
Negative	14	10 (8.70%)	4 (3.48%)	
Positive	101	48 (41.74%)	53 (46.09%)	
Edmonson grade				0.158
II	64	28 (24.35%)	36 (31.3%)	
III	27	14 (12.17%)	13 (11.3%)	
IV	24	16(13.91%)	8 (7%)	

Table S3. The correlation between Uridine level and clincopathological features in HCC.

Clinical Variables	No. of Patients	Uridine Level		*p* Value
	n=46	High (n = 23)	Low (n = 23)	
Gender				0.665
Female	6	2 (4.3%)	4 (8.7%)	
Male	40	21 (45.7%)	19 (41.3%)	
Age(years)				0.236
<60	25	10 (21.7%)	15 (32.6%)	
≥60	21	13 (28.3%)	8 (17.4%)	
AFP(ng/ml)				0.038
>400	22	7 (15.2%)	15 (32.6%)	
≤400	24	16 (34.8%)	8 (17.4%)	
ALT(U/L)				0.376
>40	22	13 (28.3%)	9 (19.6%)	
≤40	24	10 (21.7%)	14 (30.4%)	
AST(U/L)				0.236
>40	25	15 (32.6%)	10 (21.7%)	
≤40	21	8 (17.4%)	13 (28.3%)	
Child-Pugh Class				**0.011**
A	31	20 (43.5%)	11 (23.9%)	
B	15	3 (6.5%)	12 (26.1%)	
Vascular invasion				**0.008**
No	22	16 (34.8%)	6 (13.0%)	
Yes	24	7 (15.2%)	17 (37.0%)	
Microvascular invasion				0.491
No	35	19 (41.3%)	16 (34.8%)	
Yes	11	4 (8.7%)	7 (15.2%)	
TNM stage				**0.016**
I-II	27	18 (39.1%)	9 (19.6%)	
III-IV	19	5 (10.9%)	14 (30.4%)	
Tumor size(cm)				0.373
≤5	20	8 (17.4%)	12 (26.1%)	
>5	26	15 (32.6%)	11 (23.9%)	
Tumor number				0.187
Multiple	6	5 (10.9%)	1 (2.2%)	
Single	40	18 (39.1%)	22 (47.8%)	
Tumor encapsulation				0.231
No	27	16 (34.8%)	11 (23.9%)	
Yes	19	7 (15.2%)	12 (26.1%)	
Tumor differentiation				0.136
Poor	20	7 (15.2%)	13 (28.3%)	
Well	26	16 (34.8%)	10 (21.7%)	
HBsAg				1.000
Negative	13	7 (15.2%)	6 (13%)	
Positive	33	16 (34.8%)	17 (37%)	
Edmonson grade				0.798
II	31	15 (32.6%)	16 (34.8%)	
III	12	7 (15.2%)	5 (10.9%)	
IV	3	1 (2.2%)	2 (4.3%)	
